# Evaluating Adherence to National Standards: A Two-Year Audit of Consent, Clinical Efficacy, and Longitudinal Monitoring in Electroconvulsive Therapy

**DOI:** 10.1192/bjo.2026.11175

**Published:** 2026-06-30

**Authors:** Kai Scott-Bridge, Tatenda Ruwizhu, Nusrat Nyan, Ann Blackburn, Velusamy Sivakumar

**Affiliations:** Sheffield Health Partnership University NHS Foundation Trust, Sheffield, United Kingdom

## Abstract

**Aims:**

This audit evaluated the Sheffield Health Partnership University NHS Foundation Trust’s (SHPU) adherence to standards for patients receiving Electroconvulsive Therapy (ECT) as outlined by the Electroconvulsive Therapy Accreditation Service (ECTAS) guidelines. The primary objective was to assess the documentation of consent, capacity, target symptoms, and cognitive side effects throughout the treatment course and follow-up phases. Secondary objectives were to identify any improvement in compliance compared with previous audits, and assess whether high quality ECT was being delivered, as determined by statistical analysis of changes in measures of target symptoms and cognitive side effects.

**Methods:**

A retrospective audit was conducted on all patients who underwent ECT between December 2022 and December 2024 (N=16). Data were extracted from electronic records to measure compliance with standards for documentation of consent and capacity, and completion of validated scales including the Montgomery–Åsberg Depression Rating Scale (MADRS), Mini-Addenbrooke’s Cognitive Examination (mini-ACE), Clinical Global Impression (CGI), and Item 17 of the Comprehensive Psychopathological Rating Scale (CPRS). Bonferroni corrected paired t-tests were performed on mean assessment scores pre- and post-ECT.

**Results:**

Demographics were comparable to previous datasets. Documentation of legal framework and consent prior to each treatment met SHPU/ECTAS standards. Baseline monitoring achieved 100% compliance for all measures but declined during the treatment phase, falling below 75% by course completion. One-month follow-up showed minor improvement in adherence, followed by successive reductions in months two and three. Analysis of missing data found patients receiving inpatient ECT had more missing data per person on average (11.5 vs. 8.2 for outpatient treatment). Our findings suggest similar compliance to monitoring compared with previous local and national audits. Locally delivered ECT remains efficacious: mean MADRS scores reduced from 42.1 to 21.3 (p<0.01), and mean CGI scores improved from 5.5 to 2.5 (p<0.01). Objective cognitive scores (mini-ACE) significantly increased from a mean of 19.7 to 23.9 (p<0.01), and subjective memory measures (CPRS) showed no statistically significant change.

**Conclusion:**

Findings show that while the service delivers clinically effective treatment with robust initial safeguarding, longitudinal monitoring remains inconsistent. Proposed interventions include the ECT team assuming responsibility for post-treatment monitoring to minimise variability driven by inpatient pressures/discharges to community teams during follow up, updates to local ECT documentation processes to minimise missing data, and establishing ‘ECT Champions’ within referring teams to improve communication and follow-up adherence.

